# 297. Secondary Bacterial Blood Stream Infections in Patients Hospitalized with COVID-19

**DOI:** 10.1093/ofid/ofac492.375

**Published:** 2022-12-15

**Authors:** Fareeha Adnan, Mariam A Khan, Amna Umer, Nazia Khursheed

**Affiliations:** The Indus Hospital, Karachi, Sindh, Pakistan; The Indus Hospital, Karachi, Sindh, Pakistan; The Indus Hospital, Karachi, Sindh, Pakistan; The Indus Hospital, Karachi, Sindh, Pakistan

## Abstract

**Background:**

Nosocomial Bloodstream infection (nBSI) in COVID 19 patients is an emerging clinical concern for physicians. In this study, we aimed to assess the prevalence of BSI in COVID-19 patients admitted to our hospital, The Indus Hospital, Karachi, Pakistan.

**Methods:**

This retrospective study included the RT-PCR confirmed COVID-19 patients admitted to our hospital from March 2020 to December 2021. Demographics, the incidence of BSI, frequency of pathogens from the positive blood cultures, antibiotics resistance pattern, and frequency of Multidrug-Resistant Organisms (MDROs) were obtained from the hospital’s electronic medical record. Blood cultures were performed using BD BACTEC. Bacterial identification was done by using Analytical Profile Index(API) whereas antimicrobial susceptibility testing of isolates was performed by the Kirby-Baur disk diffusion method and VITEK® 2 COMPACT.

**Results:**

Our data showed that 25% of the patients admitted to our hospital developed BSI. The incidence of BSI was higher in males than in females (62.6% vs 37.4%) and most of the patients were in the age group of 46-60 years (n=93). Gram-negative pathogens were pre-dominantly identified, (50.67%), followed by yeast (12.9%), and Gram-positive pathogens (11.8%). Among these isolates, *Acinetobacter sp.* was the most commonly identified pathogen. Figure 1 shows the incidence of BSI and Figure 2 shows the distribution of all the pathogens identified from the positive blood cultures. Antibiotics resistance pattern showed a higher prevalence of MDROs. Among the MDROs, *Acinetobacter sp.* was highest in number (97.6%), followed by 66.6% of *Pseudomonas,* 63% of *E.coli*, 62% of *S. aureus,* 61.5% of *Enterococcus,* and 50% of *Klebsiella sp*.

**Figure:**
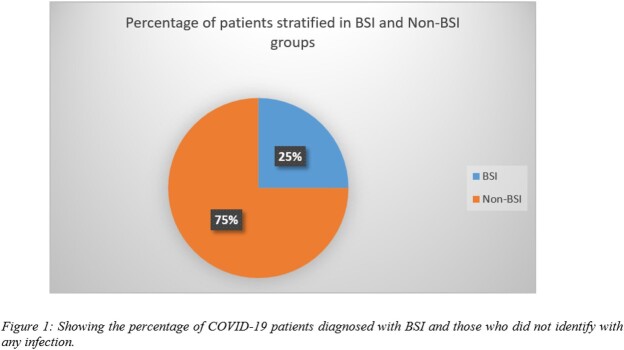

**Figure:**
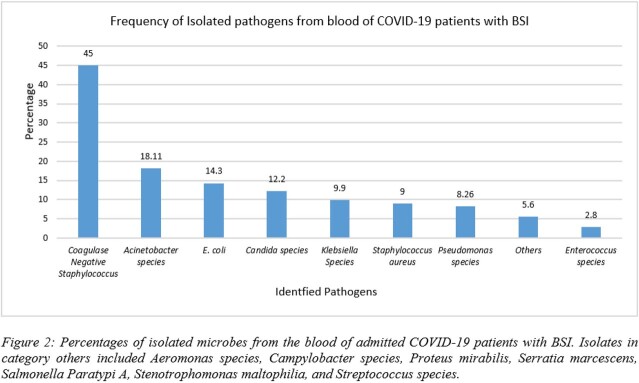

**Conclusion:**

Our findings indicated the incidence of BSI in 25% of COVID-19 in-patients. There is a significant prevalence of MDROs among which compelling prevalence of *Acinetobacter sp.* was observed. The COVID-19 pandemic overburdened the already vulnerable health care systems and made it difficult to adhere to infection control practices leading to the emergence of MDROs. Moreover, timely initiation of empirical antibiotics could also reduce the incidence of BSI in patients. Lastly, further multi-centered studies are needed for the evaluation of the incidence of BSI in our region.

**Disclosures:**

**All Authors**: No reported disclosures.

